# A genetic analysis of 23 Chinese patients with hemophilia B

**DOI:** 10.1038/srep25024

**Published:** 2016-04-25

**Authors:** Qing-Yun Wang, Bei Hu, Hui Liu, Liang Tang, Wei Zeng, Ying-Ying Wu, Zhi-Peng Cheng, Yu Hu

**Affiliations:** 1 Institute of Hematology, Union Hospital, Tongji Medical College, Huazhong University of Science and Technology, Wuhan, Hubei, 430022, China; 2 Collaborative Innovation Center of Hematology, Union Hospital, Huazhong University of Science and Technology, Wuhan, 430022, China

## Abstract

Hemophilia B (HB) is an X-linked recessive bleeding disorder caused by mutations in the coagulation factor IX (FIX) gene. Genotyping patients with HB is essential for genetic counseling and provides useful information for patient management. In this study, the *F9* gene from 23 patients with HB was analyzed by direct sequencing. Nineteen point mutations were identified, including a novel missense variant (c.520G > C, p.Val174Leu) in a patient with severe HB and a previously unreported homozygous missense mutation (c.571C > T, p.Arg191Cys) in a female patient with mild HB. Two large *F9* gene deletions with defined breakpoints (g.10413_11363del, g.12163_23369del) were identified in two patients with severe HB using a primer walking strategy followed by sequencing. The flanking regions of the two breakpoints revealed recombination-associated elements (repetitive elements, non-B conformation forming motifs) with a 5-bp microhomology in the breakpoint junction of g.12163_23369del. These findings imply that non-homologous end joining and microhomology-mediated break-induced replication are the putative mechanisms for the deletions of the *F9* gene. Because the g.12163_23369del deletion caused exons to be absent without a frameshift mutation occurring, a smaller FIX protein was observed in western blot analyses.

The human coagulation factor IX (FIX) gene, mapped at chromosome Xq27.1-q27.2, is approximately 32.7 kilobases (kb) in length. This gene contains eight exons encoding a 2.8 kb mRNA, 1.4 kb of which is translated to synthesize the vitamin K-dependent human FIX protein^1^,^2^. Heterogeneous mutations in the *F9* gene lead to deficiency or dysfunction of Factor IX and result in an X-linked inherited bleeding disorder known as hemophilia B (HB), which primarily affects approximately 1 in 25,000 male live births^3^ and very rarely affects females. Based on the activity level of FIX, HB is classified as severe (<1% of normal), moderate (1–5% of normal), or mild (5–40% of normal)^4^. The mutations associated with mild, moderate, and severe phenotypes are distributed evenly throughout the *F9* gene^5^. Currently, more than 1000 unique variants in the *F9* gene have been identified worldwide, among which 73% are point mutations, 16.3% are deletions and the remainder are insertions, duplications, or combinations of insertions and deletions (indels)^6^.

Large deletions (>50 bp) in the *F9* gene, 90% of which are associated with the severe phenotype, occur in approximately 5% of patients with severe HB and significantly increased risks for developing inhibitors^5^,^7^,^8^. Although approximately 100 types of large deletions of the *F9* gene have been reported, only 19 have defined breakpoints^9^,^10^,^11^. It has been proposed that the underlying mechanisms may be non-allelic homologous recombination (NAHR), non-homologous end joining (NHEJ) or microhomology-mediated break-induced recombination (MMBIR) events^10^. In this paper, we identified a homozygous point mutation in a female patient with mild HB. We also discovered a novel point variant and characterized the breakpoints of two suspected large deletions of the *F9* gene from a cohort of 23 patients with HB, which enabled us to explore the underlying mechanisms involved in these deletions and their roles in HB.

## Materials and Methods

### Subjects

This study was approved by the Ethics Committee of Union Hospital at Huazhong University of Science and Technology, and the methods were applied in accordance with the approved guidelines. Written informed consent was obtained from all of the participants and their respective family members. A cohort of 23 unrelated patients with HB (22 males and a 3-year-old female) were enrolled in this study. Family members of one patient (CD) were also included to confirm the carrier state of his mother (CDM) and to determine the carrier status of his sister (CDS). Peripheral blood samples obtained from the subjects were centrifuged at 1600 × g for 20 min at 4 °C. The plasma samples were aliquoted and stored in polypropylene tubes at −80 °C, and genomic DNA was extracted from the cell pellet according to the protocol provided by the manufacturer (Bioteke, Beijing, China) and as previously described^11^. The activity of FIX and other coagulation factors was assayed using a STA-R automated coagulation analyzer (Diagnostica Stago Inc., Asnieres, France) and commercial reagents from Stago according to the manufacturers’ recommendations.

### Genetic analysis and sequencing

All exons, 5’- and 3’-untranslated regions (UTRs), exon-intron junction regions and the promoter of *F9* were amplified by polymerase chain reaction (PCR). We used the primers and PCR conditions as previously reported by Hinks *et al.*^12^ The PCR products were analyzed by direct DNA sequencing using an ABI PRISM 3730XL DNA sequencer (Applied Biosystems, Carlsbad, CA, USA). Large deletions were suspected in two patients with severe HB due to partial exons repeatedly failing to be amplified from the *F9* sequences. Specifically, exon 2 and exon 3 did not amplify from the *F9* sequence of the first patient (LXF), whereas exon 4 and exon 5 did not amplify in the other patient (CD). The deletions were validated by long-range PCR (LR-PCR) using Long PCR Enzyme Mix (Life Technologies, Grand Island, NY, USA) according to the manufacturer’s two-step cycling protocol (Table S1).

To identify the junctions of the deletions, sequential primers were designed at the interval of approximately 0.5–1 kb to amplify the sequences (fragment by fragment, and the ends of the adjacent fragments were overlapping) on both sides of the breakpoints (from exon 1 to exon 4 in patient LXF and from exon 3 to exon 6 in patient CD) to narrow down the regions containing the breakpoints. The PCR products were electrophoresed on 1% agarose gel stained with GELVIEW (Bioteke, Beijing, China) for 85 min. Two pairs of sequencing primers were designed to sequence through the breakpoint (Table S1).

The nomenclature of the *F9* mutations was based on cDNA reference sequence NM_000133.3 and protein reference sequence NP_000124.1 in accordance with the recommendations of the Human Genome Variation Society (HGVS; http://www.hgvs.org/mutnomen/) guidelines.

### Bioinformatics analysis

The conservation of amino acids affected by the novel missense mutations of *F9* was analyzed by multiple sequence alignment (HomoloGene; http://www.ncbi.nlm.nih.gov/sites/entrez) in orthologous FIX sequences from human, chimpanzee, Rhesus monkey, dog, mouse, and Norway rat. The possible pathogenicity of a newly identified missense mutation was evaluated using the following *in silico* bioinformatics tools: PolyPhen-2 (http://genetics.bwh.harvard.edu/pph2/), MutationTaster (http://www.mutationtaster.org/), and Sorting Intolerant From Tolerant (SIFT; http://sift.jcvi.org)^13^,^14^,^15^. To evaluate and score the potential splice site gain or loss in mutations, the following online software tools were applied: SplicePort (http://spliceport.cbcb.umd.edu), ASSP (http://wangcomputing.com/assp/index.html), and BDGP (www.fruitfly.org/seq_tools/splice.html). To investigate the presence of certain sequence features surrounding the breakpoint regions (300 bp down- and upstream from the breakpoint junction), a series of bioinformatics analyses were performed to determine the presence of repeat sequences, sequence motifs associated with DNA breakage and sequences leading to non-B DNA conformations that predispose carriers to genomic rearrangements, as previously described^10^,^16^.

### Western blotting, ELISA and thrombin generation assays

To evaluate the influence of the large deletions on plasma factor IX protein and blood coagulation, western blotting, ELISA and a thrombin generation test were performed using plasma samples from patient LXF and patient CD, and from CDM and CDS. CDS served as a control because she had no identified mutations and normal factor IX activity. Plasma samples, 15 μl, from LXF, CD, CDM and CDS were electrophoresed on 10% polyacrylamide gels with SDS and were blotted onto a polyvinylidene difluoride membrane. The primary antibody was mouse monoclonal antibody (Santa Cruz Biotechnology, Dallas, TX, USA) specific for an epitope mapping between amino acids 301 and 339 within an internal region of FIX of human origin. GAPDH served as the control. The blots were analyzed using the ECL system.

Quantification of plasma FIX levels was performed using a ZYMUTEST FIX ELISA kit (Hyphen BioMed, Andresy, France), according to the manufacturer’s protocol.

The thrombin generation in platelet poor plasma (PPP) was measured using a calibrated automated thrombogram (Thrombinoscope BV, Maastricht, the Netherlands), according to the manufacturer’s instructions. Briefly, 80-μL plasma samples were mixed with 20 μL PPP reagent (5 pM tissue factor, 4 μM phospholipids) in a 96-well plate. Coagulation was initiated by adding 100 mM calcium chloride (20 μL) in a custom BSA buffer containing 2.5 mM fluorogenic substrate (Z-Gly-Gly-Arg-AMC). As a result of splitting by thrombin, the fluorescent AMC (7-amino-4-methylcoumarin) was released and measured using a 390-nm excitation and a 460-nm emission filter set in an Ascent Fluoroscan. Fluorescence was recorded for 60 min.

## Results and Discussion

### FIX activity and mutation profile

**Figure 1:**
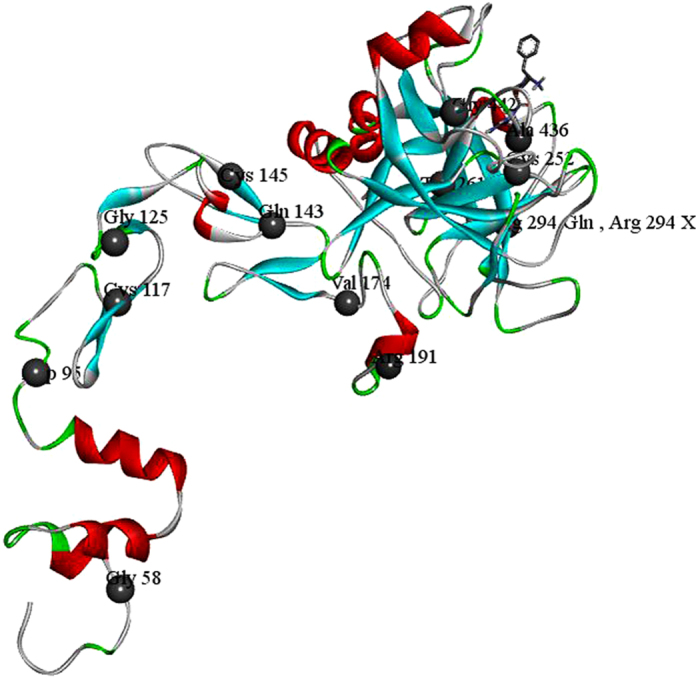
Crystal structure of factor (F) IXa and locations of the missense mutations. Ribbon view of the human FIXa structure to show the locations of the missense mutations. The domain orientation is taken from the porcine FIXa crystal structure (PDB ID: 1PFX). The alpha helix is colored in red, the beta strand in cyan, the band in grey and turn in green.

DNA sequencing has been increasingly performed and applied to assess genetic disorders because the process has become less expensive and more convenient. Routine causative mutation screening by direct sequencing in patients with HB is essential for genetic counseling and prenatal diagnosis. A total of 23 patients with HB and family members of patient CD (n = 2) were genotyped by direct sequencing in the current study. Of these patients, 8 suffered from severe HB, 11 suffered from moderate HB and 3 suffered from mild HB. The severity of one patient with HB was unknown because his factor IX activity was not detected. Factor IX activity of patient CD’s family members was 47% (CDM) and 87% (CDS) of normal. Nineteen unique point mutations in the *F9* gene were identified, including a novel missense variant (c.520G > C, p.Val174Leu) from a patient with severe HB and a previously unreported homozygous point mutation (c.571C > T, p.Arg191Cys) that was identified from the *F9* gene sequence of a 3-year-old female patient with mild HB (Table 1, Fig. 1). In one patient with mild HB, we failed to identify any *F9* gene variants. The reason may be that potential variations could exist in introns or promoter regions that our sequencing did not cover. As previously described, another likelihood is that the patient might possess somatic mosaicisms and that the DNA extracted for genotyping was derived from normal blood cells^17^. Large deletions were suspected in two patients with severe HB because the partial exons of the *F9* gene repeatedly failed to be amplified (Table 1). Consistent with the factor IX mutation database^5^,^6^, 76.2% of the mutations identified in the current study were missense mutations, and 8.7% were gross deletions (>50 bp). No inhibiting antibodies to the factor IX protein were detected in any of the patients with HB in our study.

### Novel *F9* missense variants

Of the 19 point mutations in our study, one missense variant (c.520G > C, p.Val174Leu) was novel and associated with severe HB and one homozygous (c.571C > T, p.Arg191Cys) missense variant associated with mild HB was previously unreported. Both of the variants could not be found in 1000 Genomes Browser (http://www.ncbi.nlm.nih.gov/variation/tools/1000genomes/), indicating that there was an extremely small likelihood that the variants were prevalent HB-neutral single nucleotide polymorphisms. The amino acid p.Val174 affected by missense mutations is located in the linker region of Factor IX and is highly conserved in chimpanzee, Rhesus monkey, dog, mouse, Norway rat, chicken and frog. Different point mutations at the same site (c.520G > A, p.Val174Met and c.520G > T, p.Val174Leu) have been reported^6^. The three subjects affected by the mutations at this site had moderate to severe disease; thus indicating that mutations at this site have detrimental consequences.

Because the full-length *F9* transcript has been shown to be difficult to obtain from peripheral lymphocytes^18^ and *in silico* analysis had been demonstrated to be valuable in achieving accurate predictions^19^, it would be informative to analyze the mutations affecting splice sites using web tools. Therefore, considering the nucleotide substitution (c.520G > C) at the 3’-end of exon 5, the impact of this variant on the splice site was analyzed using three bioinformatics tools. The score values obtained from the mutated sequences were low, suggesting that the splice donor site could be altered by the variant, resulting in abnormal alternative splicing and a frameshift in the FIX protein product. Hence, this could likely be the underlying mechanism that might explain why the patient affected by the variant c.520G > C suffered severe HB. But, as the online prediction tools are not very reliable, the exact mechanism remains to be clarified.

Although identifying the base substitution c.571C > T in the *F9* gene was not novel^8^, the homozygous mutation of c.571C > T identified from the 3-year-old female patient with HB was previously unreported. The probability that the amino acid change (p.Arg191Cys) is deleterious was high at 1.000 based on the PolyPhen-2 and SIFT analyses, indicating that the mutation causes the Factor IX protein dysfunction and is harmful to the patient. These results might imply that the patient has severe HB. However, the activity level of FIX in her plasma sample was 19.5% of normal. The discrepancy between what was expected and what the activity level actually was may be explained by the activation process of FIX. The FIX protein is activated by either factor VIIa/tissue factor (FVIIa/TF) or Factor XIa (FXIa) through a process that requires the removal of the activation peptide^20^,^21^, which is accomplished through two proteolytic cleavages: first, an Arg191-Ala192 bond between the light chain and the activation peptide (AP) is cleaved, generating an inactive two-chain intermediate held together by a disulfide bond. Second, an Arg226-Val227 bond between the AP and catalytic domain is cleaved, thus releasing the AP and producing the final product, FIXa^22^. The mutation p.Arg191Cys disables the activation peptide from being cleaved from the light chain of FIX, which may influence the normal structure of the enzyme and the binding affinity of FIX with its substrate. However, because the Arg226-Val227 bond could be cleaved, the catalytic domain was not influenced. Consequently, the abnormal factor IX protein in the plasma sample of the young female patient still had some catalytic activity. Owing to the unreliability of PolyPhen-2 and SIFT and the effects of the variant was unproven without expression studies, additional research on the effect of the variant is warranted.

### Characterization of the large deletions of the *F9* gene

**Figure 2:**
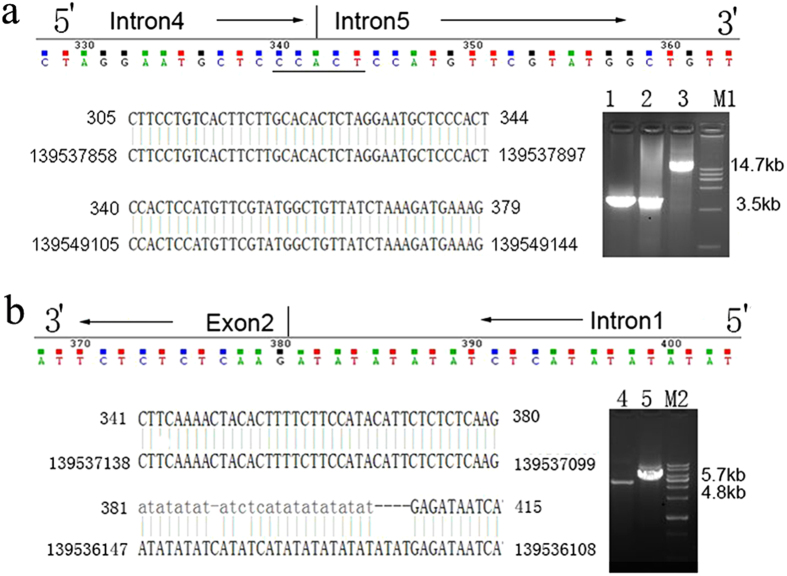
Identification of the breakpoints from two patients with severe hemophilia B. Figure 2a,b represent patients CD and LXF, respectively. Lanes 1, 2, 3, 4 and 5 represent the DNA sequences amplified from CD, CDM, CDS, LXF, and normal control, respectively. M1and M2 are DL15000 DNA Marker (Solarbio, Beijing, China) and 1 kb DNA Ladder (Solarbio, Beijing, China), respectively. The reference sequence used for the DNA sequence alignment is NCBI NC_000023.11.

In our study, the large *F9* gene deletions identified in two patients with severe HB (patient CD and LXF) were confirmed using long-range PCR; each of the fragments amplified from the hemophilic subjects’ and CDM’s (CDM, mother of the patient CD) genomic DNA samples was shorter than that of the control (Fig. 2). Considering that the factor IX activity of the asymptomatic CDM was 47% of normal, it was interesting that only a shorter fragment was amplified from her *F9* sequence, which indicated that CDM could be a heterozygous carrier and that the shorter fragment could be prior to amplify. The size of the fragment amplified from CDS (sister of patient CD) was as large as predicted, suggesting that CDS was normal. The flanking regions surrounding the junctions were narrowed. Sequential primers were used to amplify the fragments on both sides of the breakpoints toward the junctions. The breakpoints were finally defined by direct sequencing using two pairs of sequencing primers. The large deletion mutations were g.10413_11363del and g.12163_23369del. The extents of the deletions of the two patients were 953 bp and 11,207 bp, respectively.

The flanking sequences surrounding the junctions were analyzed using online bioinformatics tools. The repetitive elements were enriched at only one flanking side of the breakpoint junction of the two large deletion mutations. The 5’ flanking region of the breakpoint junction was mapped within a DNA/TcMar-Tigger repeat in patient LXF. The breakpoint of patient CD resided in the LINE/L1 repeat and nonrepeat sequences, respectively, and was associated with a 5-bp microhomology (Table S2). *In silico* analysis showed that the breakpoint-flanking regions contained multiple noncanonical DNA structures and were not enriched with GC content or palindromes. Direct repeats, potentially leading to slipped hairpin structures, were found in both patients. Mirror repeats, potentially leading to triplex structures, and inverted repeats, leading to a cruciform structure, were only identified in the flanking regions of patient LXF. Neither G-rich sequences for forming G-quadruplex structures nor left-handed Z-DNA forming regions were found in the breakpoint regions of the patients (Table S2).

The deletions of the chromosomal segments (copy number variants, CNVs), an underlying factor in many diseases, are not randomly distributed in the human genome but tend to be clustered in regions of the complex genomic architecture^23^. The potential mechanisms mediating the recombination of deletions in the *F9* gene had been described by Wu *et al.*^10^ Consistent with the findings in a previous study^10^,^16^, the deletion with a 5-bp microhomology at the breakpoint junction in the *F9* gene of patient CD can be explained by both NHEJ and MMBIR mechanisms. In patient LXF, there was no microhomology in the junction. The deletion might be mediated by the NHEJ mechanism. Naturally occurring repetitive DNA sequences can adopt non-B DNA structures and can often co-localize with chromosomal breakpoint “hot-spots”^24^,^25^. The genes harboring repetitive DNA sequences and non-B DNA structure-forming sequences increase the risk of genetic instability and are associated with large deletions of the *F9* gene.

### Western blotting, ELISA and thrombin generation test

**Figure 3:**
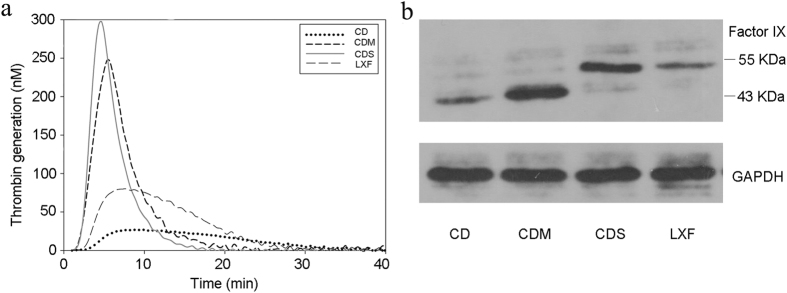
Thrombin generation test and western blotting.

The 5’-end and 3’-end of deletions in patient LXF were in intron 1 and exon 2 of the *F9* gene, respectively, which suggested that the normal intron 1 acceptor splice site was absent from the *F9* gene. The junctions were also analyzed using bioinformatics tools. The score value for patient LXF was below the software detection thresholds, indicating that there was no new splice site forming at the junction, resulting in a frameshift mutation. No splice site was affected in patient CD. The deletions of exon 4 and exon 5 predicted an in-frame shortened protein with loss of all EGF1 and EGF2 domain residues. The results from the western blots confirmed that only a normal-sized factor IX species was presented in the plasma sample of LXF as a result of prophylactic therapy. The plasma sample from CD, however, showed a normal-sized factor IX species from his prophylactic therapy and a smaller species corresponding to his hemophilic factor IX, which was predicted to be ~9 kd lower in molecular weight. The plasma sample from CDM also showed both a normal-sized factor IX species and a ~9 kd smaller species, indicating that both of the normal and truncated factor IX existed in the plasma sample of CDM (Fig. 3). Our results also suggested that the frameshift mutation in LXF might introduce a premature stop codon, leading to an abnormal polypeptide chain translated, which could degrade quickly or could not conjugate with the primary antibody used in this study. The deletions of exon 4 and exon 5 in patient CD and in CDM were also confirmed using western blots, resulting in an in-frame shortened protein without all EGF1 and EGF2 domain residues. FIXa interacts with FVIIa/TF using the EGF1 domain, and it binds to platelets and assembles with the FX activating complex using the EGF2 domain^26^,^27^. Therefore, the FIX protein without EGF1 and EGF2 domain in carriers could not function normally, causing severe HB of male carriers.

Compared with the control, the plasma factor IX antigen of the patients was severely reduced. The FIX antigen levels of LXF, CD and CDM were reduced to 1.64%, 0.78% and 54.81% level of control, respectively. Because prophylactic infusions of the factor IX concentrate were used in both hemophilic patients, we thought that it would be more meaningful to compare the plasma factor IX antigen level and the ability of the thrombin generation between CDM and CDS. The FIX antigen level of CDM was reduced to 54.81% of the control; however, the endogenous thrombin potential of CDM was equal to that of CDS. The ability of the thrombin generation was significantly different between the patients with HB and the normal control. The main parameters, including the lag time, the area under the curve (ETP) and the peak of the patients, were much lower than those of the control (Fig. 2).

In conclusion, 22 different mutations of the *F9* DNA sequence were identified from 23 unrelated patients with HB in this study, 4 of which were novel, including two large *F9* gene deletions. Furthermore, we pinpointed and characterized the large deletions, implicating NHEJ and MMBIR as the underlying recombination mechanisms mediating their formation. This study revealed the role of genetic variations in human disorders and highlighted the role that genomic instability may play in predisposition to the formation of pathogenic imbalances.

## Additional Information

**How to cite this article**: Wang, Q.-Y. *et al.* A genetic analysis of 23 Chinese patients with hemophilia B. *Sci. Rep.* **6**, 25024; doi: 10.1038/srep25024 (2016).

## Supplementary Material

Supplementary Information

## Figures and Tables

**Table 1:** Summary of the 19 different *F9* gene mutations identified in 23 unrelated patients with hemophilia B.

P	FIX:C (%)	FIX:Ag (%)	Nucleotide change	CpG	AA substitution	Region	Domain
1	1	96	c.1324G > A	N	p.Gly442Arg	E 8	SP
2	0.8	<1	c.391 + 1G > C		–	I 4	–
3	2	2.3	c.881G > A	Y	p.Arg294Gln	E 8	SP
4	0.6	<1	c.520G > C*	N	p.Val174Leu	E 5	Linker
5	0.8	1.8	g.10413_11363del*			E 2 & I 1	GLA/PP
6	1	47	c.1307C > A	N	p.Ala436Gln	E 8	SP
7	0.7	110	c.677G > A	N	p.Arg226Gln	E 6	AP
8	1	1.3	c.349T > C	N	p.Cys117Arg	E 4	EGF1
**9**	5.1	87	c.172G > C	N	p.Gly58Arg	E 2	GLA
10	1.8	2.3	c.880C > T	Y	p.Arg294X	E 8	SP
11	0.2	76	c.676C > T	Y	p.Arg226Trp	E 6	AP
12	–	–	c.434G > A	N	p.Cys145Tyr	E 4	EGF2
13	3.7	–	c.374G > A	N	p.Gly125Glu	E 4	EGF1
14	0.4	<1	c.59T > C	N	p.Leu20Ser	E 1	Signal Peptide
15	2.6	1.4	c.781T > C	N	p.Trp261Arg	E 7	SP
16	1.5	67	c.427C > G	N	p.Gln143Glu	E 5	EGF2
**17**	1	<1	c.172G > C	N	p.Gly58Arg	E 2	GLA
18	3.9	20	c.284A > G	N	p.Asp95Gly	E 4	EGF1
19	1.9	2.1	c.755G > A	N	p.Cys252Tyr	E 7	SP
20	<1	<1	c.252 + 1G > A	N	–	I 2	–
21	6.2	–	–	–	–	–	–
22	19.5	120	c.571C > T	Y	p.Arg 191 Cys	E 6	Linker
23	<1	<1	g.12163_23369del*	–	–	E 4 & E 5	EGF1&2
CDM	47	59	g.12163_23369del*	–	–	E 4 & E 5	EGF1&2
CDS	87	108	–	–	–	–	–

^*^Novel mutations; P, patients; AP, act-peptide; SP, serine protease; E, exon; I, intron; CDM, mother of patient 23; CDS, sister of patient 23.
