# Supplementary material for: A genetic analysis of 23 Chinese patients with hemophilia B
Source: Sci Rep. 2016 Apr 25;6:25024. doi: 10.1038/srep25024 (PMC4842959; doi:10.1038/srep25024)
Supplement: Supplementary Information [file srep25024-s1.doc]

**Supplemental data**

**Title page**

A genetic analysis of 23 Chinese patients with hemophilia B

Qing-Yun Wang,^1,2#^ Bei Hu,^1,2#^ Hui Liu,^1#^ Liang Tang,^1,2*^ Wei Zeng,^1,2^ Ying-Ying Wu,^1^ Zhi-Peng Cheng,^1,2^ and Yu Hu^1,2*^

^1^Institute of Hematology, Union Hospital, Tongji Medical College, Huazhong University of Science and Technology, Wuhan, Hubei, 430022, China;

^2^Collaborative Innovation Center of Hematology, Union Hospital, Huazhong University of Science and Technology, Wuhan, 430022, China

^#^ The first three authors contributed equally to this work

Address correspondence to: Yu Hu

Institute of Hematology, Union Hospital, Tongji Medical College, Huazhong University of Science and Technology, Wuhan, 430022, China; Tel.: +86 27 85726335; fax: +86 27 85726387.

E-mail: [dr_huyu@126.com](mailto:dr_huyu@126.com)

Or Liang Tang

Institute of Hematology, Union Hospital, Tongji Medical College, Huazhong University of Science and Technology, Wuhan, 430022, China; Tel.: +86 27 85726335; fax: +86 27 85726387.

E-mail: [lancet.tang@qq.com](mailto:lancet.tang@qq.com)

**Acknowledgments:** This study was supported by grants from the National Natural Sciences Foundation of China (No. 81370622 and No. 81400099)

**Competing financial interests declaration:** The authors declare they have no actual or potential competing financial interests

**Table S1.**

**PCR primers and conditions used for charactering the deletions of the factor IX gene**

| **Primer** | **Forward sequence (5′→3′)** | **Reverse sequence (5′→3′)** | **Annealing temp. (°C)** | **Product size (bp)** |
| --- | --- | --- | --- | --- |
| CD-LR | AACATCACAGATTTTGGCTCCATGCCCTAA | GACCTTCATGTTGTTGACCAGTTGCCTGTC | 68 | 14676 |
| CD-1 | GAAGCACGAGAAGTTTTTGA | TAGTTCCCACACTGGCATAA | 54 | 429 |
| CD-2 | TGGAAGCAGTATGTTGGTAAGC | CTGGGGCAGGTTGAAGTATTA | 58 | 675 |
| CD-3 | GGCTTTATCTGGCTGTTTC | GCTGGTATGGTAGTATTCGTTT | 50 | 742 |
| CD-4 | CTCATAACTCCTCCACTTTTG | AGATGCTAGTAATGTGGTTGG | 50 | 630 |
| CD-5 | CTGTACCATCAACTCATCACA | CCGTTGACTAAGAGACTAATATC | 50 | 797 |
| CD-6 | TTAGTCTCTTAGTCAACGGAAC | GTCTTGTAGAGCAAACTGAGC | 50 | 783 |
| CD-7 | TGTACTGAGGGATATCGACTT | TCTTTAGATAACAGCCATACG | 50 | 698 |
| CD-8 | ACATCTCCACTCCATGTTCGT | GCAGCACTGTGATATGCTCGT | 58 | 671 |
| CD-9 | GTAAACCCACAACCTTCCAGTGT | TCAAGCCACATTCCTTGTAACAG | 58 | 637 |
| CD-10 | GTCTGTTACAAGGAATGTGGC | CAGCACGGGTGAGCTTAG | 55 | 797 |
| CD-Seq | TGAATGGTGATATACTACAGGGTTATGCC | TGCTGAGACAGGTACTGAAACAGTTACAG | 68 | 11805 |
| LXF-LR | TGGCTTCTAAAAGGAGTTTCGGTGAGTGAT | AGAGTGGTAAGGCTACTGGGGCAGGTTGA | 68 | 5734 |
| LXF-1 | TAAACTCTCATTGGCTTC | CTCTCTCCTAGTCTTGGTA | 50 | 740 |
| LXF-2 | CAGTCTGGCAACACGCTA | CTATTCTGGTTCCCCGCT | 55 | 990 |
| LXF-3 | GGTTCACTCTTGTCCCAG | TCCATTTACAGTCGTTCC | 50 | 992 |
| LXF-4 | GTAAGCCATTTTTATATCGG | GAAGTTGCCTGGAGTTGT | 50 | 995 |
| LXF-5 | GCAACTTCTTGAGTCCCT | TTTGGTTGGCAAGTGTTA | 50 | 797 |
| LXF-6 | CCTATAACACTTGCCAACC | TTACCAACATACTGCTTCC | 50 | 933 |
| LXF-7 | TGGAAGCAGTATGTTGGTAA | TGGGGCAGGTTGAAGTAT | 55 | 674 |
| LXF-Seq | TAGGTTGTATTGATGTGGCCTATTGC | GGACTGATCTTTCTGAGTCCTTTTGAC | 65 | 1612 |

CD and LXF stand for two patients with severe hemophilia B; LR, primers for long-range polymerase chain reaction; Seq, primers for sequencing; 1...N, represent primers used for amplifying the flanking sequences of the breakpoints.

**Table S2．** Microhomology, repetitive elements and non-B DNA-forming motifs in the flanking regions of the deletions.

| **Patients** | **MH (bp)** | **Repetitive elements** | | **Non-B DNA-forming motifs** | | | | | **Potential**  **mechanism** |
| --- | --- | --- | --- | --- | --- | --- | --- | --- | --- |
|  |  | **Proximal** | **Distal** | **DR** | **IR** | **MR** | **GS** | **Z-DNA** |  |
| CD | 5 | LINE/L2 | - | + | - | - | - | - | NHEJ/MMBIR |
| LXF | - | DNA/TcMar-Tigger | - | + | + | + | - | - | NHEJ |

MH, Microhomology; -, not found; +, observed; LINE, long interspersed nuclear element; DR, Direct repeats; IR, IR, inverted repeats; MR, mirror repeats; IR, inverted repeats; MR, mirror repeats; GS, G-quadruplex structure; NHEJ, non-homologous end joining; MMBIR, microhomology-mediated break-induced recombination.
